# Patients With Myasthenia Gravis With Acute Onset of Dyspnea: Predictors of Progression to Myasthenic Crisis and Prognosis

**DOI:** 10.3389/fneur.2021.767961

**Published:** 2021-11-18

**Authors:** Yangyu Huang, Ying Tan, Jiayu Shi, Ke Li, Jingwen Yan, Yuzhou Guan

**Affiliations:** Department of Neurology, Peking Union Medical College Hospital, Peking Union Medical College, Chinese Academy of Medical Sciences, Beijing, China

**Keywords:** myasthenia gravis, myasthenic crisis, impending myasthenic crisis, early-onset, intravenous immunoglobulin, mechanical ventilation, post-intervention status

## Abstract

**Background:** Life-threatening myasthenic crisis (MC) occurs in 10–20% of the patients with myasthenia gravis (MG). It is important to identify the predictors of progression to MC and prognosis in the patients with MG with acute exacerbations.

**Objective:** This study aimed to explore the predictors of progression to MC in the patients with MG with acute onset of dyspnea and their short-term and long-term prognosis.

**Methods:** This study is a retrospective cohort study. We collected and analyzed data on all the patients with MG with acute dyspnea over a 10-year period in a single center using the univariate and multivariate analysis.

**Results:** Eighty-six patients with MG were included. In their first acute dyspnea episodes, 36 (41.9%) episodes eventually progressed to MC. A multivariate analysis showed that the early-onset MG (adjusted *OR*: 3.079, 95% *CI* 1.052–9.012) and respiratory infection as a trigger (adjusted *OR*: 3.926, 95% *CI* 1.141–13.510) were independent risk factors for the progression to MC, while intravenous immunoglobulin (IVIg) treatment prior to the mechanical ventilation (adjusted *OR*: 0.253, 95% *CI* 0.087–0.732) was a protective factor. The prognosis did not significantly differ between the patients with and without MC during the MG course, with a total of 45 (52.3%) patients reaching post-intervention status better than minimal manifestations at the last follow-up.

**Conclusion:** When treating the patients with MG with acute dyspnea, the clinicians should be aware of the risk factors of progression to MC, such as early-onset MG and respiratory infection. IVIg is an effective treatment. With proper immunosuppressive therapy, this group of patients had an overall good long-term prognosis.

## Introduction

Myasthenia gravis (MG) is an autoimmune disease caused by the autoantibodies that affect the structure of the post-synaptic membrane. Its main clinical manifestation is fluctuating muscle weakness (1). Myasthenic crisis (MC) refers to an event that requires mechanical ventilation because of severe involvement of the bulbar muscles or/and respiratory muscles (2). Furthermore, 10–20% of the patients with MG will develop MC (3–6), and most MC occurs within 2 years of the onset of MG (6, 7). IVIg and plasma exchange are recommended as the first-line therapy for impending and manifest myasthenic crisis (2, 8–10). However, the proportion of Chinese patients using IVIg and plasma exchange is significantly lower than that of the United States, and an Indian study on MC mentioned they treat the patients with MC with only corticosteroids and other immunosuppressive agents (11), suggesting that in the developing countries, many patients are not treated adequately due to the high price and unavailability of IVIg and plasma exchange. Therefore, exploring the risk factors of progress to MC in the patients with acute onset of dyspnea will aid clinical decision-making and medical resource allocation, especially in the developing countries. However, the previous studies focused on the clinical characteristics of the patients with MC (4, 12, 13) or the risk factors of MC occurrence after thymectomy (14–16), very few studies aimed to investigate the predictors of progression to MC in the patients with MG with acute onset of dyspnea. This study aimed to explore the predictors of progression to MC in the patients with MG with acute onset of dyspnea and their short-term and long-term prognosis.

## Methods

### Study Design and Patient Selection

This study is a retrospective cohort study. We included all the patients with MG who complained of acute dyspnea and visited the emergency department of the Peking Union Medical College Hospital, Beijing, China from September 2010 to June 2020. The diagnosis of myasthenia gravis was made by satisfying the following three criteria: (1) fluctuating muscle weakness; (2) positive neostigmine test, or decrements of compound motor action potential showed in slow repetitive nerve stimulation, or positive serum anti-acetylcholine receptor (AChR) antibody; (3) other causes of skeletal muscle weakness excluded. The patients with dyspnea occurring within 4 weeks after thymectomy, or positive muscle-specific tyrosine kinase (MuSK) antibodies, or dyspnea because of acute exacerbation of chronic cardiopulmonary disease were excluded from this study. MC (2) was defined as receiving mechanical ventilation (non-invasive or invasive ventilation) or arterial blood gas analyses suggesting that the indications for mechanical ventilation were met (PaCO_2_ > 45 mmHg and pH < 7.35, or oxygenation index PaO_2_/FiO_2_ < 200) (17). Multiple emergency department visits of the same patient were analyzed separately. We recommend IVIg for all the patients complaining of dyspnea. When mechanical ventilation was indicated, non-invasive ventilation was used first if the conditions permitted. All the patients were discharged from the hospital with oral corticosteroids and/or immunosuppressive agents. The final treatment choices were based on the informed consents of the patients and their family members. This study was approved by the Ethics Committee of Clinical Research of Peking Union Medical College Hospital (Beijing, China). The informed consents were obtained from every patient.

### Data Acquisition

We collected data of all acute dyspnea episodes and selected the first episodes of each enrolled patient to explore the predictors of progression to MC. For predictive variables, we retrospectively collected demographic data, clinical features of MG, and comorbidities by reviewing the medical record data. All the patients underwent chest CT or contrast-enhanced chest CT, and the conditions of thymus were documented according to imaging or pathology. We obtained the data on the triggers of dyspnea, AChR antibody status, the Myasthenia Gravis Foundation of America (MGFA) clinical classification at the last follow-up before the episode, the treatments before the dyspnea episode, and the specific treatments (IVIg and glucocorticoid impulse therapy) used before the progression to crisis. For the outcome variables, the primary outcome was the occurrence of MC, the secondary outcomes included short-term prognosis and long-term prognosis, the former including the type and length of mechanical ventilation, total length of hospital stay, and in-hospital complications, and the long-term prognosis included the post-intervention status (PIS) at the last follow-up.

### Statistics

The numerical variables were summarized as mean ± SD or median (inter quartile range, IQR), respectively, depending on whether they followed normal distribution. For univariate analysis, *t*-test or Wilcoxon's rank sum test was used for group comparisons of numerical variables, and chi-square test or Fisher's exact test was used for the categorical variables. Significance level α was set at 0.05 for both the sides. To find independent risk factors for progression to MC in the patients with acute onset of dyspnea, first dyspnea episodes of each patient were divided into two groups according to whether MC occurred, and the distribution of each predictor variable was compared between the two groups using a univariate analysis. All the variables that achieved *p* < 0.10 were selected for the bivariate logistic regression model. If multiple selected variables were correlated clinically (e.g., age of onset and early onset MG), only one variable of them was selected for the multivariate analysis. Thereafter, the *p*-values and adjusted *OR* values of the selected variables were calculated using the bivariate logistic regression. A principal component analysis was preformed to evaluate and eliminate multicollinearity. The data analysis was conducted using the IBM-SPSS (version 25, IBM, NY, USA).

## Results

### Baseline Characteristics

We collected the clinical data from 86 patients with MG who had 111 visits to the emergency department for acute onset of dyspnea (Table 1). In total, 20 (23.3%) patients experienced multiple episodes of dyspnea (maximum of four episodes). Thirty-six (41.9%) of the patients were male. Of the 86 first dyspnea episodes of each patient, 35 (40.7%) occurred in the patients with thymoma and 23 (26.7%) in the patients who had undergone thymectomy. The mean age of MG onset was 47.2 ± 17.8 years, 45 (52.3%) dyspnea episodes occurred in the patients with early-onset MG (i.e., age of onset not older than 50), and 62 (72.1%) episodes occurred in the patients with MG with ocular onset. Fifty-seven (66.3%) patients had positive AChR antibody, while 22 (25.6%) patients lacked the data. The median time from MG onset to dyspnea episode was 12.0 (4.0–34.5) months, and the median age of dyspnea episode was 51.0 ± 17.1. We divided the skeletal muscles other than respiratory muscles into four groups: ocular muscles, limb muscles, bulbar muscles, and cervical muscles. Thirty (34.9%) dyspnea episodes occurred in the patients with involvement of all the four muscle groups, while 60 (69.8%) episodes in the patients with involvement of three or more groups. We also collected MGFA clinical classification at the last follow-up before the episode, and 53 (61.6%) dyspnea episodes occurred in the patients with MGFA clinical classification ≥3a at the last follow-up. Fifty-eight (67.4%) dyspnea episodes occurred in the patients who were not on corticosteroids or other immunosuppressive agents, while 56 (65.1%) episodes occurred in the patients who had not used corticosteroids before. Twenty-one (24.4%) episodes occurred after a trigger of respiratory infection, while other triggers included long-time fatigue, other infections, and other unspecified predisposing factors. In 55 (64.0%) dyspnea episodes, the patients received IVIg prior to mechanical ventilation. Regarding the comorbidities, the most frequent comorbidity was diabetes mellitus (14 episodes, 16.3%), followed by other autoimmune diseases (10 episodes, 11.6%). Thirty-six (41.9%) dyspnea episodes eventually progressed to MC, and 32 patients received mechanical ventilation, 28 (87.5%) of which ended with invasive mechanical ventilation.

**Table 1:** The baseline characteristics of first acute dyspnea episodes of the patients with myasthenia gravis (MG) and comparison between myasthenic crisis (MC) and non-MC episodes.

	**All episodes**	**MC episodes**	**Non-MC episodes**	***p*-value**
	**(86 episodes)**	**(36 episodes)**	**(50 episodes)**	
Female, *n* (%)	50 (58.1)	21 (58.3)	29 (58.0)	0.975
Thymoma, *n* (%)	35 (40.7)	17 (47.2)	18 (36.0)	0.296
Thymectomy, *n* (%)	23 (26.7)	11 (30.6)	12 (24.0)	0.498
Age of MG onset, mean ± SD	47.2 ± 17.8	41.1 ± 17.7	51.6 ± 16.7	0.006^*^
Early onset MG, *n* (%)	45 (52.3)	24 (66.7)	21 (42.0)	0.024^*^
AChR antibody status, *n* (%)				0.406
Positive	57 (66.3)	19 (52.8)	38 (76.0)	
Negative	7 (8.1)	4 (11.1)	3 (6.0)	
Unknown	22 (25.6)	13 (36.1)	9 (18.0)	
Involved muscle groups at MG onset				0.163
Ocular muscles, *n* (%)	62 (72.1)	23 (63.9)	39 (78.0)	
Limb muscles, *n* (%)	10 (11.6)	6 (16.7)	4 (8.0)	
Bulbar muscles, *n* (%)	12 (14.0)	5 (13.9)	7 (14.0)	
Cervical muscles, *n* (%)	2 (2.3)	2 (5.6)	0	
Respiratory muscles, *n* (%)	0	0	0	
Time from MG onset to dyspnea episode (month), median (IQR)	12.0 (4.0–34.5)	9.5 (4.3–33.0)	12.0 (2.0–37.5)	0.759
Age of dyspnea onset, mean ± SD or median (IQR)	51.0 ± 17.1	44.7 ± 17.0	60.5 (46.7–66.0)	0.004^*^
Involved muscle groups at dyspnea onset, *n* (%)
Ocular muscles	81 (94.2)	31 (86.1)	50 (100.0)	0.025^*^
Limb muscles	66 (76.7)	27 (75.0)	39 (78.0)	0.745
Bulbar muscles	72 (83.7)	31 (86.1)	41 (82.0)	0.61
Cervical muscles	38 (44.2)	15 (41.7)	23 (46.0)	0.69
4 muscle groups involved	30 (34.9)	11 (30.6)	19 (38.0)	0.475
>2 muscle groups involved	60 (69.8)	23 (63.9)	37 (74.0)	0.314
MGFA classification at last visit before dyspnea episode, *n* (%)				0.024^*^
1	16 (18.6)	2 (5.6)	14 (28.0)	
2a	7 (8.1)	4 (11.1)	3 (6.0)	
2b	4 (4.7)	2 (5.6)	2 (4.0)	
3a	14 (16.3)	2 (5.6)	12 (24.0)	
3b	24 (27.9)	14 (38.9)	10 (20.0)	
4a	9 (10.5)	4 (11.1)	5 (10.0)	
4b	6 (7.0)	3 (8.3)	3 (6.0)	
Unknown	6 (7.0)	5 (13.9)	1 (2.0)	
MGFA classification worse than 3a, *n* (%)	53 (61.6)	23 (63.9)	30 (60.0)	0.714
Treatment before dyspnea episode, *n* (%)				0.603
No immunosuppressive treatments	58 (67.4)	23 (63.9)	35 (70.0)	
Steroids	19 (22.1)	8 (22.2)	11 (22.0)	
Other immunosuppressants alone	1 (1.2)	0	1 (2.0)	
Steroids and other immunosuppressants	8 (9.3)	5 (13.9)	3 (6.0)	
Respiratory infection as the trigger of the dyspnea episode, *n* (%)	21 (24.4)	13 (36.1)	8 (16.0)	0.037^*^
IVIg before MV, *n* (%)	55 (64.0)	17 (47.2)	38 (76.0)	0.006^*^

*AChR, anti-acetylcholine receptor; IQR, inter quartile range; IVIg, intravenous immunoglobulin, MC, myasthenic crisis; MG, myasthenia gravis; MGFA classification, Myasthenia Gravis Foundation of America clinical classification; MV, mechanical ventilation. SD, standard deviation*.

* *p < 0.05*.

Twenty (23.3%) patients experienced multiple episodes of dyspnea (maximum of four episodes), they had 45 episodes in total. Of the additional 25 episodes after the first episodes, 10 (40.0%) episodes were triggered by respiratory infection, 16 (64.0%) episodes were treated by IVIg. Ten (40.0%) episodes progressed to MC at last, similar to the first episode described above.

We analyzed the usage of IVIg at different time periods. From 2010 to 2013, IVIg was used in 20 out of 34 episodes (58.8%). This proportion was 36/57 (63.2%), 15/20 (75.0%) in different periods of 2014–2017 and 2018–2020, respectively. Although there appears to be an increasing in the use of IVIg, Wilcoxon's rank sum test did not reach statistical significance (*p* = 0.484).

### Predictors of Progression to MC

Of the 86 first dyspnea episodes of each patient, 36 (41.9%) eventually progressed to MC (Table 1). By comparing the baseline characteristics between the MC group and non-MC group using the univariate analysis, we found that the variables with *p* < 0.10 included age of MG onset (*p* = 0.006), early-onset MG (*p* = 0.024), age at the dyspnea episode (*p* = 0.009), ocular muscle involvement when dyspnea episode occurred (*p* =0.025), MGFA clinical classification at last follow-up (*p* = 0.024), respiratory infection as the trigger (*p* = 0.037), and IVIg therapy before mechanical ventilation (*p* = 0.006). Before including these variables in the binary logistic regression, we noted that age at onset correlated with early-onset or late-onset MG, and we took only early-onset MG into the regression. Thereafter, MGFA classification at last follow-up was dichotomized (≥3a vs. <3a).

The results of the bivariate logistic regression are shown in Table 2. The final equation suggested early-onset MG (adjusted *OR*: 3.079, 95% *CI* 1.052–9.012) and respiratory infection as a trigger (adjusted *OR*: 3.926, 95% *CI* 1.141–13.510) were independent risk factors for MC, while IVIg treatment prior to mechanical ventilation (adjusted *OR*: 0.253, 95% *CI* 0.087–0.732) was a protective factor for MC. Further the principal component analysis showed no covariance between the selected variables.

**Table 2:** The multivariable analysis for predictors of progression to MC.

**Predictors**	**Adjusted OR**	**95% CI of adjusted OR**	***p*-value**
Early onset MG	3.079	1.052–9.012	0.040
Respiratory infection as a trigger	3.926	1.141–13.510	0.030
IVIg treatment prior to MV	0.253	0.087–0.732	0.011

*IVIg, Intravenous immunoglobulin; MC, myasthenic crisis; MG, myasthenia gravis; MV, mechanical ventilation; OR, odds ratio*.

### Clinical Characteristics and Prognosis of Patients With and Without MC During the MG Course

Of the 20 patients with multiple dyspnea episodes, seven patients did not experience MC in their first episodes but had MC in subsequent episodes. Thus, in all 86 patients, 49 (57.0%) had no MC during the course of MG, while 37 (43.0%) had MC (Table 3). The patients with early-onset MG were more likely to develop MC (*p* = 0.025). Thymoma was more frequent in the patients experienced MC, though the statistical difference was not significant (48.6 vs. 34.7%, *p* = 0.192). Follow-up time was defined as the time from the start of the last episode to the last follow-up visit. There was a significant difference in the follow-up time between the two groups (*p* = 0.020), with the patients without MC followed for 36.5 ± 22.8 months, while the patients with MC were followed for 60.4 ± 55.2 months. The long-term prognosis was similar in both the groups, with 57.1% of the non-MC group having PIS better than minimal manifestations (MM) at the last follow-up, compared with 45.9% of patients in the MC group.

**Table 3:** The clinical characteristics and prognosis of patients with and without MC during the MG course.

	**All patients**	**Non-MC patients**	**MC patients**	***p*-value**
	**(*n* = 86)**	**(*n* = 49)**	**(*n* = 37)**	
Female, *n* (%)	50 (58.1)	30 (61.2)	20 (54.1)	0.517
Comorbidities, *n* (%)
Pulmonary disease	5 (5.8)	3 (6.1)	2 (5.4)	0.684
Cardiac disease	2 (2.3)	1 (2.0)	1 (2.7)	1
Kidney disease	1 (1.2)	1 (2.0)	0	1
Liver disease	7 (8.1)	4 (8.2)	3 (8.1)	1
Diabetes mellitus	14 (16.3)	11 (22.4)	3 (8.1)	0.074
Neoplasm other than thymoma	3 (3.5)	2 (4.1)	1 (2.7)	1
Other autoimmune disease	10 (11.6)	5 (10.2)	5 (13.5)	0.893
Thymoma, *n* (%)	35 (40.7)	17 (34.7)	18 (48.6)	0.192
Thymectomy, *n* (%)	26 (30.2)	11 (22.4)	15 (40.5)	0.071
Age of MG onset, mean ± SD	47.2 ± 17.8	52.3 ± 16.1	40.4 ± 17.9	0.002
Early onset MG, *n* (%)	45 (52.3)	20 (40.8)	25 (67.6)	0.025
Involved muscle groups at dyspnea onset, *n* (%)				0.191
Ocular muscles	62 (72.1)	38 (77.6)	24 (64.9)	
Limb muscles	10 (11.6)	4 (8.2)	6 (16.2)	
Bulbar muscles	12 (14.0)	7 (14.3)	5 (13.5)	
Cervical muscles	2 (2.3)	0	2 (5.4)	
Respiratory muscles	0	0	0	
Follow-up time (month), mean ± SD or median (IQR)	41.0 (12.8–63.5)	36.5 ± 22.8	60.4 ± 55.2	0.02
PIS at last follow up, *n* (%)				0.307
CSR	2 (2.3)	1 (2.0)	1 (2.7)	
PR	34 (39.5)	23 (46.9)	11 (29.7)	
MM	9 (10.5)	4 (8.2)	5 (13.5)	
Improved	19 (22.1)	10 (20.4)	9 (24.3)	
Unchanged	1 (1.2)	1 (2.0)	0	
Exacerbation	11 (12.8)	5 (10.2)	6 (16.2)	
Death unrelated to MG	1 (1.2)	1 (2.0)	0	
Unknown	9 (10.5)	4 (8.2)	5 (13.5)	
PIS better than MM at last follow-up, *n* (%)	45 (52.3)	28 (57.1)	17 (45.9)	0.303

*CSR, complete stable remission; IQR, inter quartile range; MC, myasthenic crisis; MG, myasthenia gravis; MM, minimal manifestations; PIS, Myasthenia Gravis Foundation of America (MGFA) post-intervention status; PR, Pharmacologic Remission; SD, standard variation*.

### Characteristics of Patients Requiring Invasive Ventilation Longer Than 15 Days

Of the 35 MC episodes requiring invasive ventilation, 12 (34.3%) had ventilation over 15 days (Table 4). One-third of them had comorbid diabetes mellitus, significantly more than the other group (*p* = 0.038). The patients requiring prolonged mechanical ventilation also had significantly more pneumonia complications (83.3 vs. 39.1%, *p* = 0.03) and longer total length of hospital stay (46.5 vs. 25.7 days, *p* = 0.003). In addition, the patients requiring prolonged mechanical ventilation had older age (51.7 ± 13.1 years) than the other group (42.9 ± 20.2 years), but no significant difference was reached (*p* = 0.13).

**Table 4:** The characteristics of patients requiring invasive ventilation longer than 15 days.

	**Invasive ventilation**	**Ventilation < 15 days**	**Ventilation > 15 days**	***p*-value**
	**(*n* = 35)**	**(*n* = 23)**	**(*n* = 12)**	
Female, *n* (%)	18 (51.4)	14 (60.9)	4 (33.3)	0.164
Comorbidities, *n* (%)
Pulmonary disease	3 (8.6)	2 (8.3)	1 (8.3)	1
Cardiac disease	1 (2.9)	1 (4.3)	0	1
Kidney disease	0	0	0	-
Liver disease	5 (14.3)	3 (13.0)	2 (16.7)	1
Diabetes mellitus	5 (14.3)	1 (4.3)	4 (33.3)	0.038
Neoplasm other than thymoma	0	0	0	-
Other autoimmune disease	6 (17.1)	3 (13.0)	3 (25.0)	0.391
Thymoma, *n* (%)	19 (54.3)	13 (56.5)	6 (50.0)	0.736
Thymectomy, *n* (%)	13 (37.1)	9 (39.1)	4 (33.3)	0.164
Age of MG onset, mean ± SD or median (IQR)	39.6 ± 18.6	31.0 (21.0–55.0)	44.4 ± 18.2	0.203
Early onset MG, *n* (%)	23 (65.7)	15 (65.2)	8 (66.7)	1
Age of dyspnea onset, mean ± SD	45.9 ± 18.3	42.9 ± 20.2	51.7 ± 13.1	0.13
Respiratory infection as the trigger of the dyspnea episode, *n* (%)	16 (45.7)	11 (47.8)	5 (41.7)	1
IVIg before MV, *n* (%)	15 (42.9)	9 (39.1)	6 (50.0)	0.721
Length of hospital stay (day), mean ± SD or median (IQR)	29.0 (7.0-560.0)	25.7 ± 14.0	46.5 (30.5-72.0)	0.003
Complications, *n* (%)
Pneumonia	19 (54.3)	9 (39.1)	10 (83.3)	0.03
Liver injury	5 (14.3)	4 (17.4)	1 (8.3)	0.64
Kidney injury	2 (5.7)	1 (4.3)	1 (8.3)	1
Cardiac arrest	2 (5.7)	0	2 (16.7)	0.111
Infection other than pneumonia	4 (11.4)	2 (8.7)	2 (16.7)	0.594

*IQR, inter quartile range; IVIg, intravenous immunoglobulin; MG, myasthenia gravis; MV, mechanical ventilation; SD, standard variation*.

## Discussion

In our present study, we demonstrated that in the patients with MG with acute onset of dyspnea, early-onset MG (adjusted *OR*: 3.079, 95% *CI* 1.052–9.012) and respiratory infection as a trigger (adjusted *OR*: 3.926, 95% *CI* 1.141–13.510) were independent risk factors for progression to MC, while the use of IVIg prior to mechanical ventilation (adjusted *OR*: 0.253, 95% *CI* 0.087–0.732) was a protective factor. The occurrence of MC had no significant impact on the long-term prognosis of these patients, and more than half of the patients (52.3%) reached a PIS better than MM at the last follow-up. Of the 35 MC episodes requiring invasive ventilation, 12 (34.3%) needed ventilation for more than 15 days. Comorbid diabetes mellitus (33.3 vs. 14.3%, *p* = 0.038) and complicated pneumonia (83.3 vs. 39.1%, *p* = 0.030) was associated with prolonged ventilation.

We found that early-onset MG was an independent risk factor for progression to MC in the patients with MG with acute onset of dyspnea (Table 2). Similar to our findings, A. Ramos-Fransi et al. (7) found that in the patients with MG with life-threatening events (i.e., MGFA class V or class IVB), early-onset MG had a longer time to weaning from ventilation, suggesting that early-onset MG may have more severe life-threatening events and worse response to the treatment. Since most patients in our study had positive AChR antibodies and the positive rate was similar between the MC and non-MC episodes, the reason for early-onset MG was a risk factor may be that in the patients with positive AChR antibodies, early-onset MG and late-onset MG are thought to differ significantly in pathogenesis. The origin of autoimmune antibodies in early-onset MG is mostly in the thymus, while the role of the thymus in the pathogenesis of late-onset MG is unclear, since no detectable inflammation was found in the thymus (1). Besides, the clinical characteristics of early-onset MG differ from of late-onset MG, one of which is that the response of early-onset MG to immunotherapy and the prognosis are worse, which may be associated with an expansion in protective immunomodulatory mechanisms, such as peripheral T-regulatory cells, in the elderly (18).

The previous studies have shown that infection is the most common trigger of MC as well as other life-threatening events in the patients with MG (3, 4, 7, 12, 19, 20). Further, our study also found that among the patients with MG with acute onset of dyspnea, respiratory infection was an independent risk factor for progression to MC. Infection may lead to exacerbation of the symptoms of bulbar palsy and respiratory muscle weakness, while the respiratory infections can aggravate ventilation dysfunction and lead to impaired air exchange, increasing the probability of mechanical ventilation. For the patients with MG with acute dyspnea with respiratory infection, close monitoring of vital signs and preparation for mechanical ventilation is particularly essential.

Coronavirus disease 2019 (COVID-19) is an infectious disease caused by the severe acute respiratory syndrome coronavirus 2 (SARS-CoV-2) virus and has become a global pandemic (21). Although there were no patients with COVID-19 in our cases, the effect of COVID-19 on the patients with MG is noteworthy in the context of pandemic. Dyspnea caused by SARS-CoV-2 infection may have a greater risk of progression to MC, as we discussed above. The patients with MG may be more vulnerable to COVID-19 due to the immunosuppressive therapies (22). However, the current evidence on the relationship between MG and COVID-19 is highly variable. Some studies suggested that COVID-19 has a limited effect on most of the patients with MG, since most of the patients in these studies do not experience exacerbations of MG, and immunosuppressive therapy is relatively safe (23–25). In contrast, other studies found a high proportion of patients with MG exacerbations requiring rescue therapy or mechanical ventilation (26, 27). High-quality studies on the relationship between COVID-19 and MC are also lacking, which may be explained by the difficulty in diagnosing MC in patients with COVID-19. Besides MC, severe COVID-19 may also lead to respiratory failure. Furthermore, many previous cases used drugs such as hydroxychloroquine and azithromycin that may exacerbate the MG symptoms or cause myopathy, further complicating the relationship between respiratory failure and MC. Further well-designed, and prospective studies are needed.

The efficacy of IVIg in the patients with MG with acute exacerbation has been demonstrated in several randomized controlled trials (8). Likewise, our study found that IVIg was a protective factor for progression to MC in the patients with acute onset of dyspnea. Since IVIg and plasma exchange are equally effective in worsening MG (9), and IVIg is easier to use, our center uses IVIg to treat this group of patients. Although the international consensus guidance (2) and multiple national guidelines (10, 28) recommended IVIg and plasma exchange as first-line therapy for impending and manifest myasthenic crisis, IVIg was not used timely in 31.0% of acute dyspnea episodes in this study. Based on our experience, we speculate that the reason for this is that IVIg is still expensive for the patients with MG in the developing countries, even when health insurance can partially cover the cost, which prevents some patients with MG with acute dyspnea from receiving timely treatment.

All the patients in our study were prescribed with oral corticosteroids and/or immunosuppressant agents after discharge. The overall prognosis was good, with 52.3% of patients having a PIS better than MM at last follow-up, with a median follow-up time of 41.0 months. There was no significant difference in the PIS at the last follow-up between the patients experienced MC and those who did not, which is similar to other studies. Sivadasan, A et al. (29) studied the patients with MC admitted to the intensive care unit. All the patients received oral corticosteroids and immunosuppressant agents and were followed for a median time of 36 months, with 67% of them reaching PIS better than MM at the last follow-up. Spillane, J et al. (20) studied the patients with MG admitted to the intensive care unit due to acute exacerbation. Most of the patients (97%) were on oral steroids and nearly half (45%) were started with other immunosuppressants. At a median follow-up time of 4 years, 19% of patients were asymptomatic at the last follow-up and 48% reached MGFA classification better than type II. Both these studies and our study suggest that the long-term use of immunosuppressive therapy after the acute phase significantly improves the long-term prognosis of patients with MG who experienced acute dyspnea.

Our study found that in the patients requiring invasive ventilation longer than 15 days, a significantly higher proportion of patients had comorbid diabetes mellitus, prolonged length of hospital stay, and complications of pneumonia. The age at onset of dyspnea was also older in this group, although it did not reach significance difference (51.7 ± 13.1 vs. 42.9 ± 20.2, *p* = 0.13). Similar to other studies, advanced age and more chronic underlying diseases were risk factors for prolonged mechanical ventilation (3, 12), suggesting that this group of patients may require better intensive care unit management and have a higher probability of tracheotomy.

Of the patients included in this study, 67.4% had not used immunosuppressive therapy, such as oral steroids or immunosuppressants before their first dyspnea episodes. Similarly, the proportion of this group of patients in other studies was 40–50% (3, 20). Several studies showed that early immunosuppressive treatment of MG reduces the probability of MC occurrence and recurrence (30). To date, several retrospective studies showed that the use of oral steroids in ocular MG could reduce the probability of progression to generalized MG (31, 32). These results suggest that the use of immunosuppressive therapy in the patients with MG may not only improve symptoms, but also act as a disease modifier to prevent the progression of the disease course in the patients with MG.

The greatest limitation of this study arises from its single-center retrospective nature. The relative rarity of MG and the low incidence of respiratory or bulbar muscle involvement in MG resulted in a small sample size, so the results may be incidental. Selection bias is inevitable in single center studies. The lower incidence of comorbidities and the higher incidence of thymoma in our patients compared with other studies may limit the generalization of the findings to all the patients. However, compared with other studies that focused mostly on the patients with MG who had already developed MC, we expanded the target population to include the patients with MG with acute onset of dyspnea (i.e., the patients may progress to MC), effectively expanded the sample size and increased the credibility of the final conclusions.

The previous studies have focused on the patients with manifest MC, and most studies have been conducted in intensive care unit settings (4, 12, 13). A strength of this study is that we described the clinical characteristics of impending the patients with MC from the emergency department, which makes our study more relevant to the clinical practice of neurologists. Nowadays, many patients with MG with acute dyspnea in the developing countries are unable to receive IVIg because of its high price and unavailability. The significance of this study is that we explored the risk factors for progression to MC in this group of patients, indicating that when treating the patients with MG with acute dyspnea, the clinicians should be more aggressive in advancing the use of IVIg to avoid MC occurrence in early-onset MG or dyspnea triggered by respiratory infection. Initiating social assistance or transferring the patients to a qualified center are effective options.

In conclusion, we demonstrated that early-onset MG and respiratory infection as a trigger were the independent risk factors for progression to MC in the patients with MG with acute onset of dyspnea, while the use of IVIg prior to mechanical ventilation was a protective factor. With proper immunosuppressive therapy, this group of patients had an overall good prognosis.

## Data Availability Statement

The raw data supporting the conclusions of this article will be made available by the authors, without undue reservation.

## Ethics Statement

The studies involving human participants were reviewed and approved by Ethics Committee of Clinical Research of Peking Union Medical College Hospital (Beijing, China). Written informed consent to participate in this study was provided by the participants' legal guardian/next of kin.

## Author Contributions

YH and YG contributed to conception and design of the study. YH, YT, JS, JY, and KL collected the clinical data. YH, YT, and KL contributed to the data analysis and interpretation. YH wrote the manuscript. YT and YG edited the manuscript. All authors read and approved the final manuscript.

## Funding

This work was supported by the National Clinical Cohort Study of Rare Diseases for key R & D Program (grant number: 2016YFC091501) and the Beijing Municipal Sciences & Technology Commission (Z181100001718145).

## Conflict of Interest

The authors declare that the research was conducted in the absence of any commercial or financial relationships that could be construed as a potential conflict of interest.

## Publisher's Note

All claims expressed in this article are solely those of the authors and do not necessarily represent those of their affiliated organizations, or those of the publisher, the editors and the reviewers. Any product that may be evaluated in this article, or claim that may be made by its manufacturer, is not guaranteed or endorsed by the publisher.
